# Supplementary material for: Electronic bandstructure and van der Waals coupling of ReSe2 revealed by high-resolution angle-resolved photoemission spectroscopy
Source: Sci Rep. 2017 Jul 11;7:5145. doi: 10.1038/s41598-017-05361-6 (PMC5506037; doi:10.1038/s41598-017-05361-6)
Supplement: Supplementary file 1 — Supplementary Information [file 41598_2017_5361_MOESM1_ESM.pdf]

Electronic bandstructure and van der Waals coupling of $ReSe_2$ revealed by high-resolution angle-resolved photoemission spectroscopy

Lewis S Hart[1], James L Webb[1], Sara Dale[1], Simon J. Bending[1], Marcin Mucha-Kruczynski[1], Daniel Wolverson[1*], Chaoyu Chen[2], José Avila[2], Maria C. Asensio[2]

[1]Centre for Nanoscience and Nanotechnology, Department of Physics, University of Bath, Bath BA2 7AY, United Kingdom
[2]Synchrotron SOLEIL, Saint Aubin, and Université Paris-Saclay, BP 48 91192 Gif-sur-Yvette, France

Supplementary information

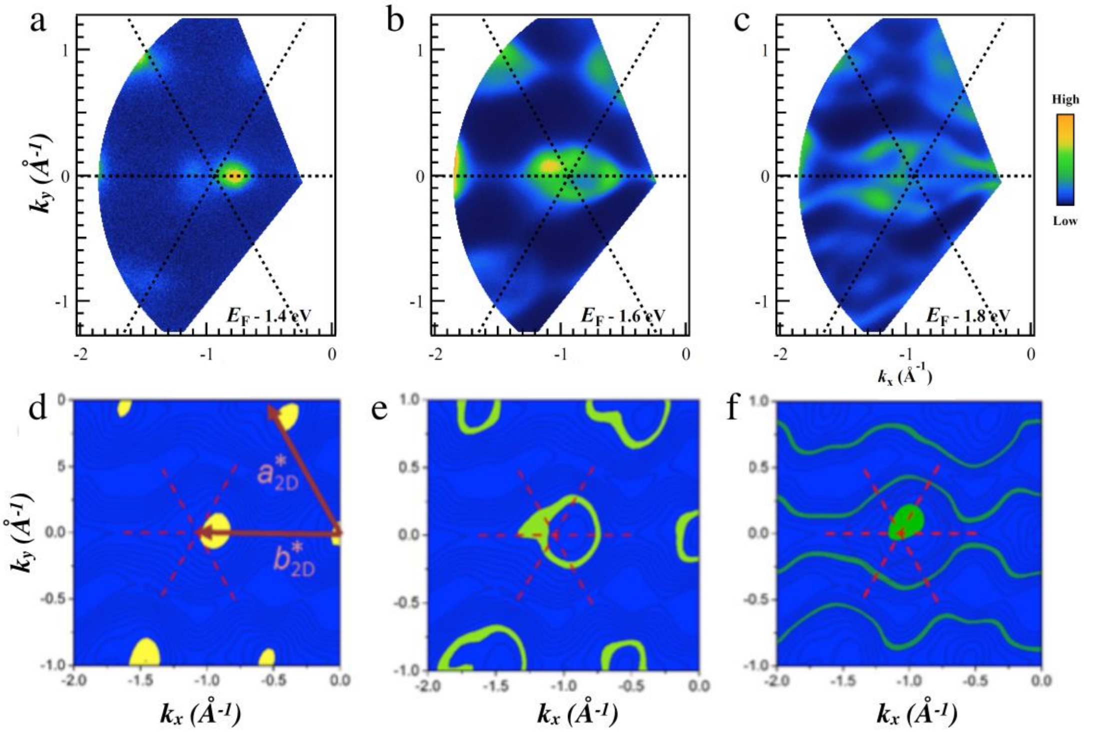

Figure S1. **ARPES maps of photoemission intensity as a function of in-plane wavevector** (**a**) near the valence band maximum (VBM); (**b**) 0.2 eV below the VBM and (**c**) 0.4 eV below the VBM. In each case, the dotted cross indicates the position of a Γ' point. For comparison, panels (**d**)-(**f**) show calculated constant energy contours of the highest energy valence band (**d**) near the VBM; (**e**) 0.2 eV below the VBM and (**f**) 0.4 eV below the VBM. In (**f**), the top of the next valence band contributes the near-central solid spot. The red arrows in (**d**) indicate the 2D projected reciprocal lattice vectors defined in Figure 1 and the dashed red lines again mark a Γ' point. The bar on the right indicates the arbitrary colour scale representing photoemission intensity (a-c), and valence band energy (d-f) respectively.

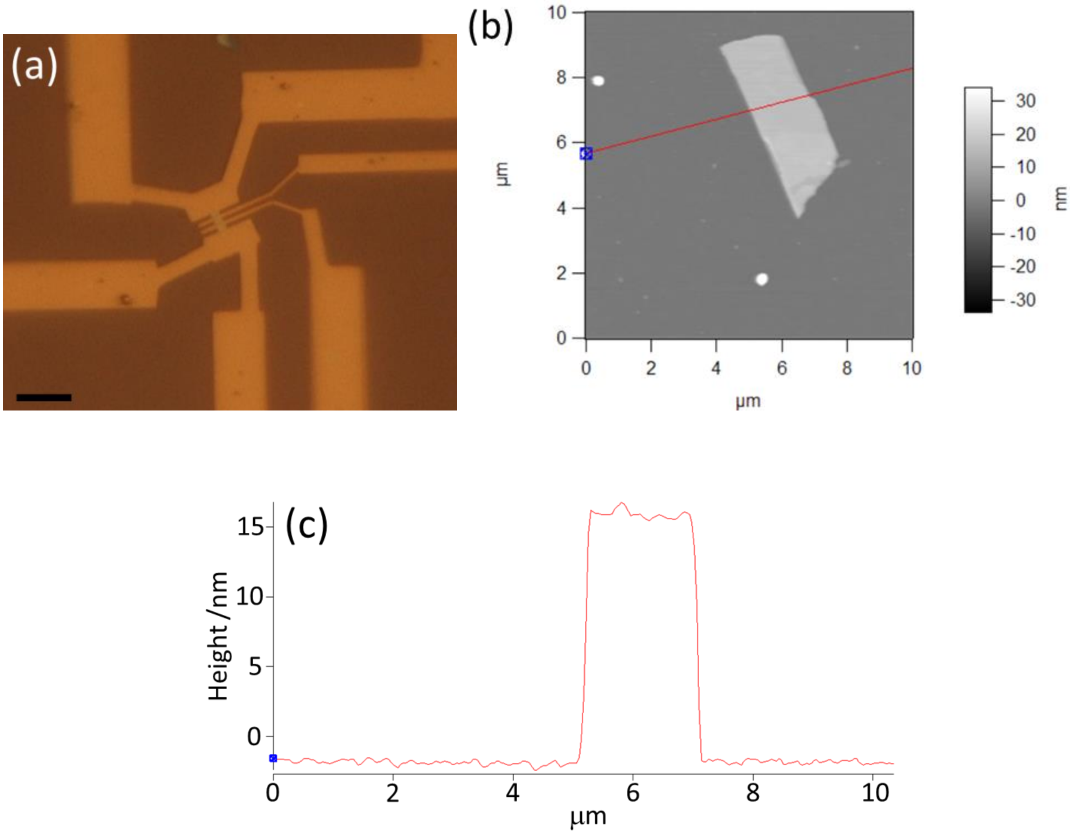

Figure S2. (a) Optical microscopy image of a field effect transistor structure fabricated from a $ReSe_2$ flake fabricated on an n^+-Si/SiO_2 (300 nm) substrate. The scale bar in bottom left hand corner represents 10 µm; (b) AFM image of the same $ReSe_2$ flake before electrical contacts were fabricated on top of the flake, with the red line showing the path of a line scan to determine the flake thickness; (c) An AFM line scan taken from image (b) to determine the height of the flake. The height was measured to be 18.3 nm and corresponds to a thickness of 26 monolayers (ref. 1 of main text).

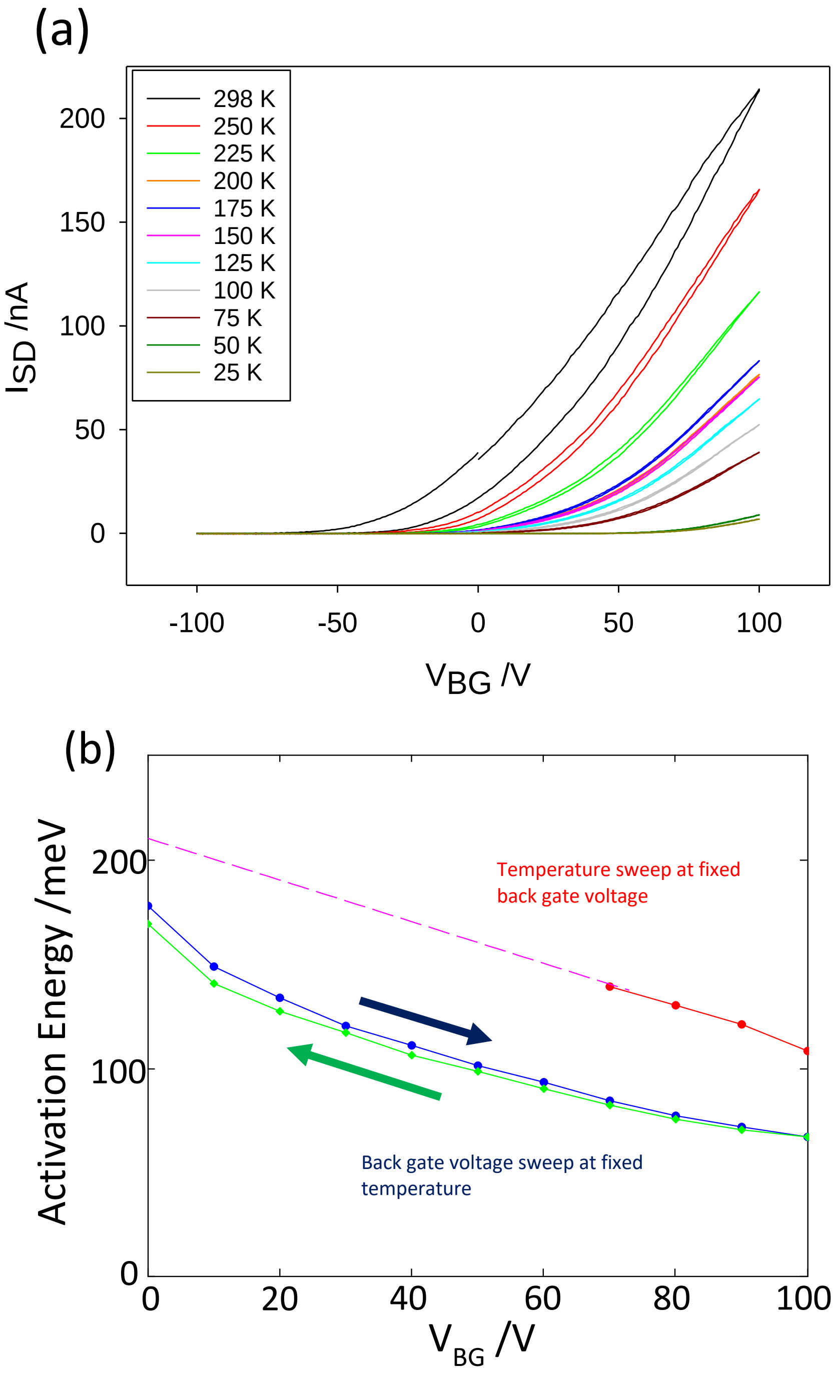

Figure S3. (a) Measurements of the $ReSe_2$ field effect transistor shown in figure S1 carried out at different temperatures with a back gate voltage V_{bg} applied to the n^+-Si substrate. The asymmetry of the source-drain current can be seen as a function of V_{bg} and clearly indicates n-type doping; (b) estimated activation energies deduced from fixed temperature back gate voltage sweeps (green and blue curves) and temperature sweep at fixed back gate voltage (red curve). A hysteresis can clearly be seen; however, both indicate an activation energy of 190 ± 20 meV, much smaller than half the band gap of $ReSe_2$.

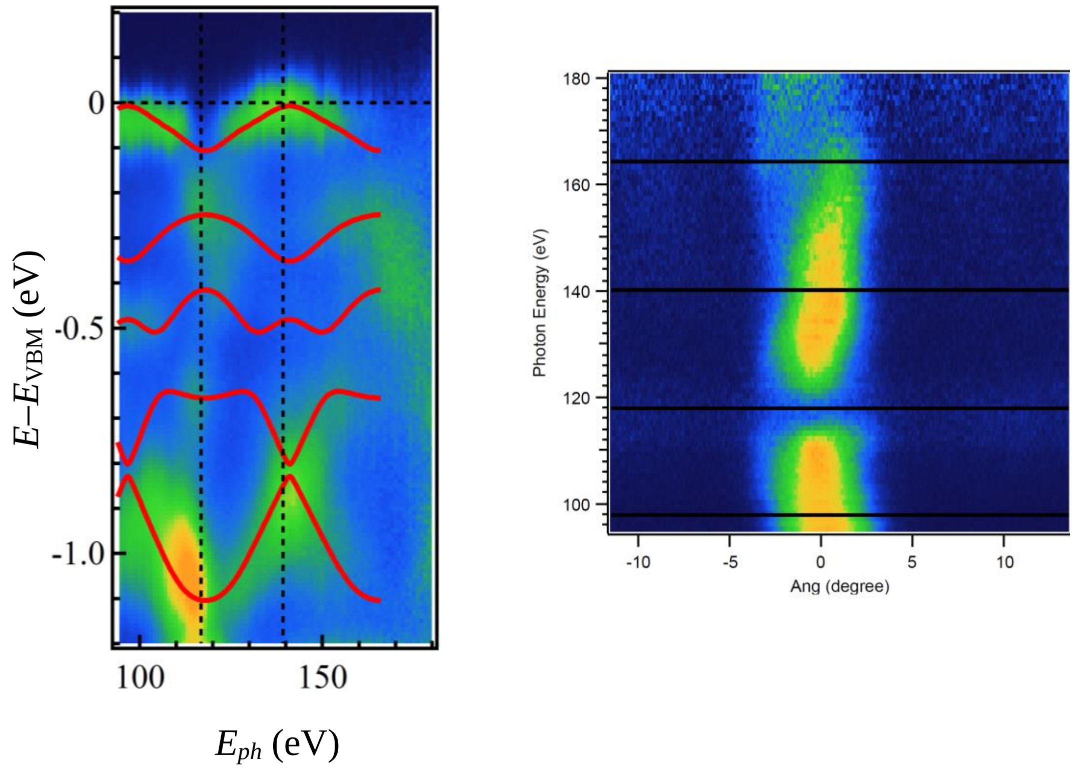

Figure S4. Left: photoemission signal for electrons with zero in-plane momentum and energies below the valence band maximum E_{VBM} as a function of excitation photon energy E_{ph}. Red lines: calculated valence band dispersion as a function of momentum in the c^* direction normal to the sample surface. Right: excerpt from the same dataset for $E\text{-}E_{VBM} = 0$, viewed as a function of emission angle to the sample normal, showing the photoemission signal for zero in-plane momentum as a function of excitation energy.

Estimate of the inner potential (V_0)

From the above data, there are minima and maxima in $E\text{-}E_{\mathrm{VBM}}$ for the top valence band at excitation energies of 118 and 141 eV. Taking equation 1 of the main text,

$$|k_z| = \frac{\sqrt{2m}}{\hbar}\sqrt{(E_{kin}\,cos^2\theta) + V_0}$$

and recognizing that k_z is only determined to within an integer multiple n of the reciprocal lattice vector in the c^* (z) direction, then we require that, for $\theta = 0$ ($k_x = k_y = 0$),

$$n|c^*| = \frac{\sqrt{2m}}{\hbar}\sqrt{E_{kin} + V_0}, \quad E_{kin} = 118 \text{ eV}$$

and

$$\left(n + \tfrac{1}{2}\right)|c^*| = \frac{\sqrt{2m}}{\hbar}\sqrt{E_{kin} + V_0}, \qquad E_{kin} = 141 \text{ eV}$$

These conditions are satisfied with $c^* = 0.984$ Å^{-1} and $V_0 = 19.1$ eV if $n = 6$. Adjacent integers (or half-integers) yield negative or implausibly large values of V_0.

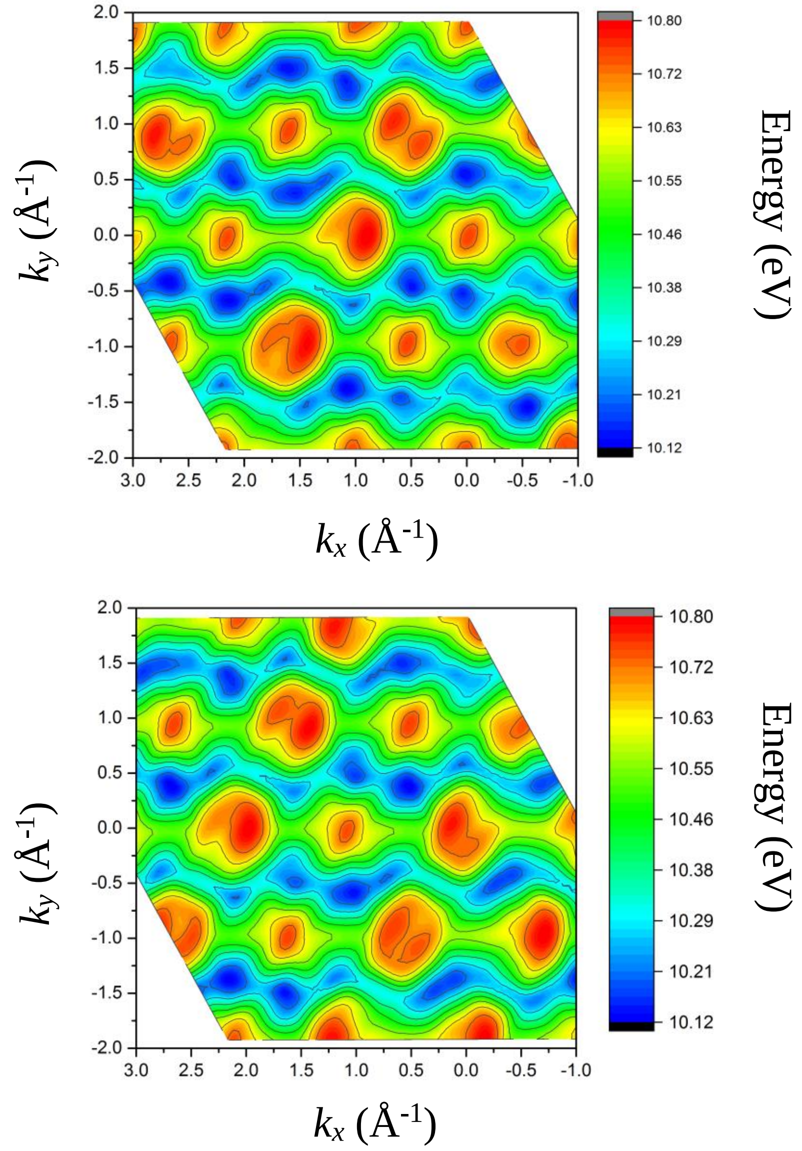

Figure S5.
Calculated ARPES sections through the highest valence band as a function of in-plane momentum k_x and k_y, taking into account the effects of the inner potential. Top: excitation energy 97 eV (which places the Z point at $k_x = k_y = 0$); bottom: excitation energy 118 eV (with the Γ point at $k_x = k_y = 0$). This demonstrates how the choice of excitation energy (and therefore c^* component) affects the measured section through the valence band constant-energy surfaces.

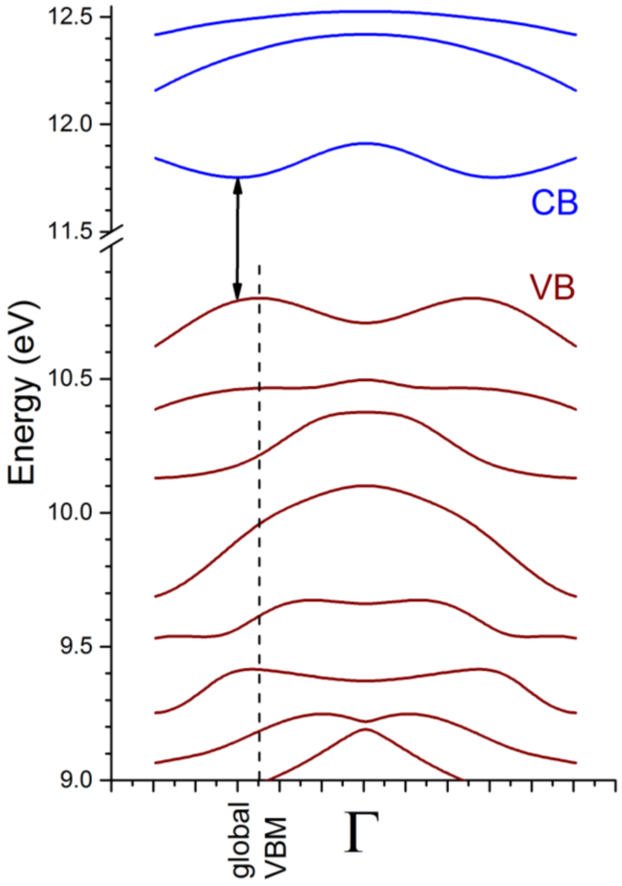

Figure S6
Calculated conduction band (top) and valence band (bottom) dispersion (energy versus momentum) for a path along the line passing through the two global valence band maxima within the first Brillouin zone (these are related by inversion through Γ). The vertical arrow through the conduction band minimum demonstrates that, at the VBM, the valence to conduction band transition is indirect though the band extrema lie quite close.

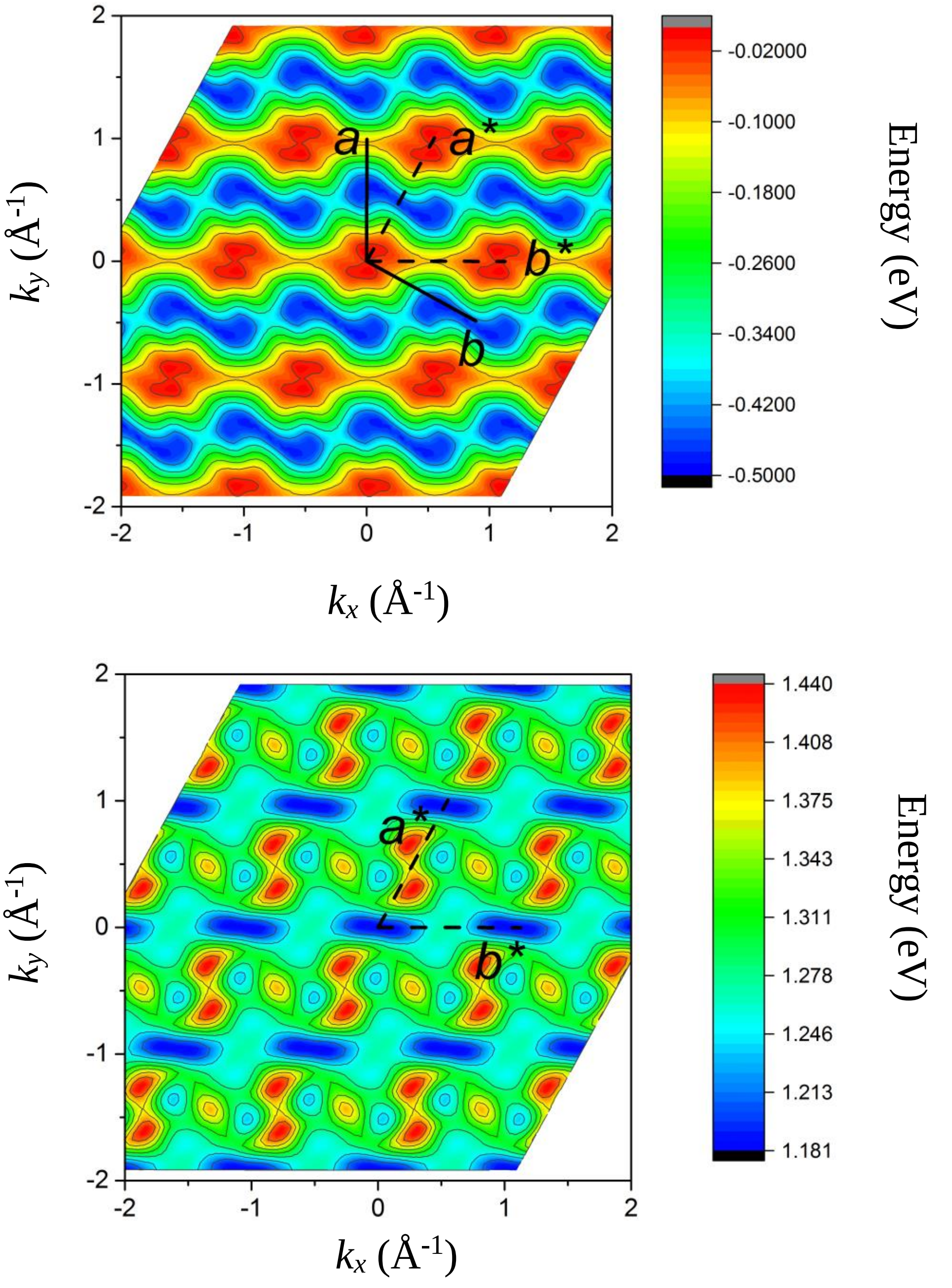

Figure S7

Calculated energies of the highest-lying valence band (top) and lowest conduction band (bottom) as a function of in-plane momenta k_x and k_y for a $ReSe_2$ **monolayer**. The reciprocal and real space lattice vectors are indicated. The local valence band maxima are highest in energy (red) and the conduction band minima are low in energy (blue), showing that the optical transition for monolayers is again indirect within the approximations of the fully relativistic DFT used (spin-orbit coupling included).

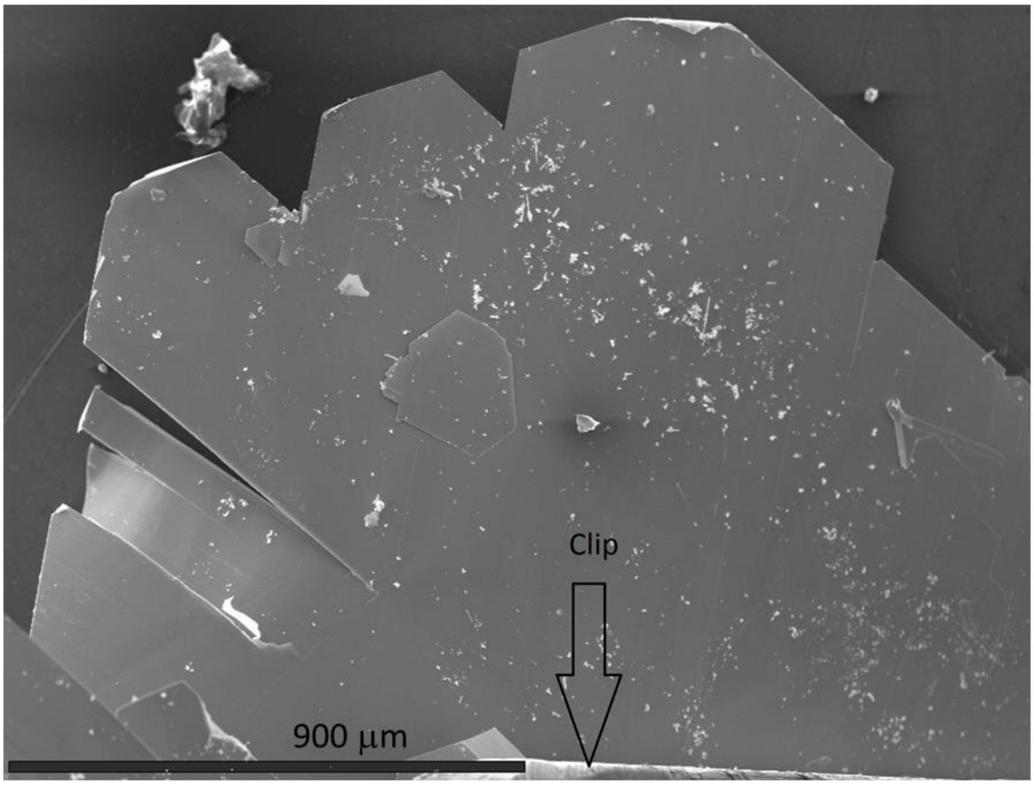

Figure S8
Scanning electron microscope image of a typical bulk crystal (which is the starting material before cleaving in UHV) showing large crystalline domains but with some misoriented detached flakes and few-micron-scale debris on the surface (scale bar is bottom left). Freshly cleaved surfaces will have less debris but a high spatial resolution was advantageous in finding regions (after cleaving in UHV) that gave well-resolved ARPES spectra associated with a single crystal domain.

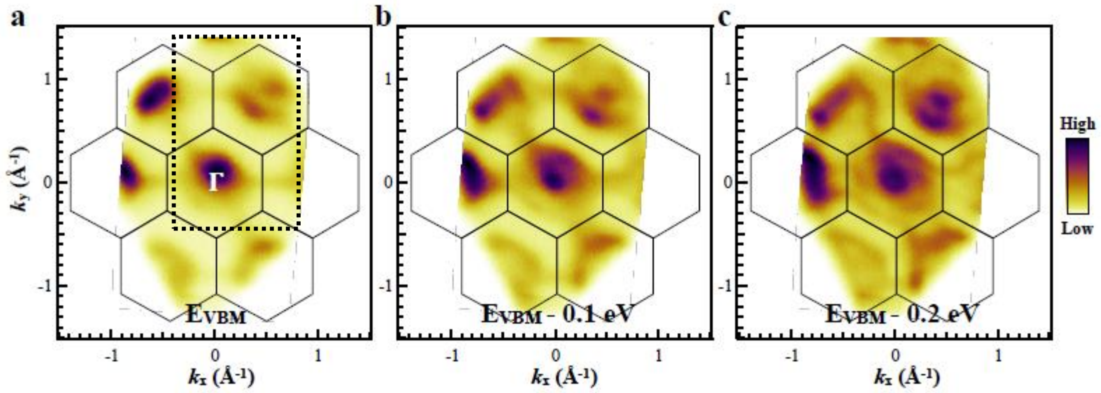

Figure S9

ARPES maps of photoemission intensity as a function of in-plane wavevector (a) near the valence band maximum (VBM); (**b**) 0.1 eV below the VBM and (**c**) 0.2 eV below the VBM. The excitation energy was 118 eV corresponding, according to the calculation of Fig. S4, to placing the three-dimensional Γ point at $k_x = k_y = 0$ in the ARPES maps. The above data can be compared to the simulation of Fig. S5, lower panel, which is reproduced again below for convenience; the dotted box in the experimental data highlights the Γ point and one neighbouring Γ' point which has a clear double structure due to the bifurcation of the constant energy surfaces shown in the main text, Fig. 5. Similar "double" Γ' points appear also in the simulated data below, and the Γ point at $k_x = k_y = 0$ shows a similar off-centre shape.

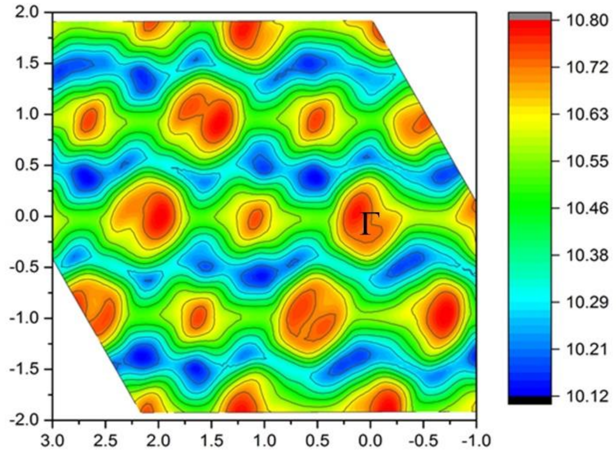

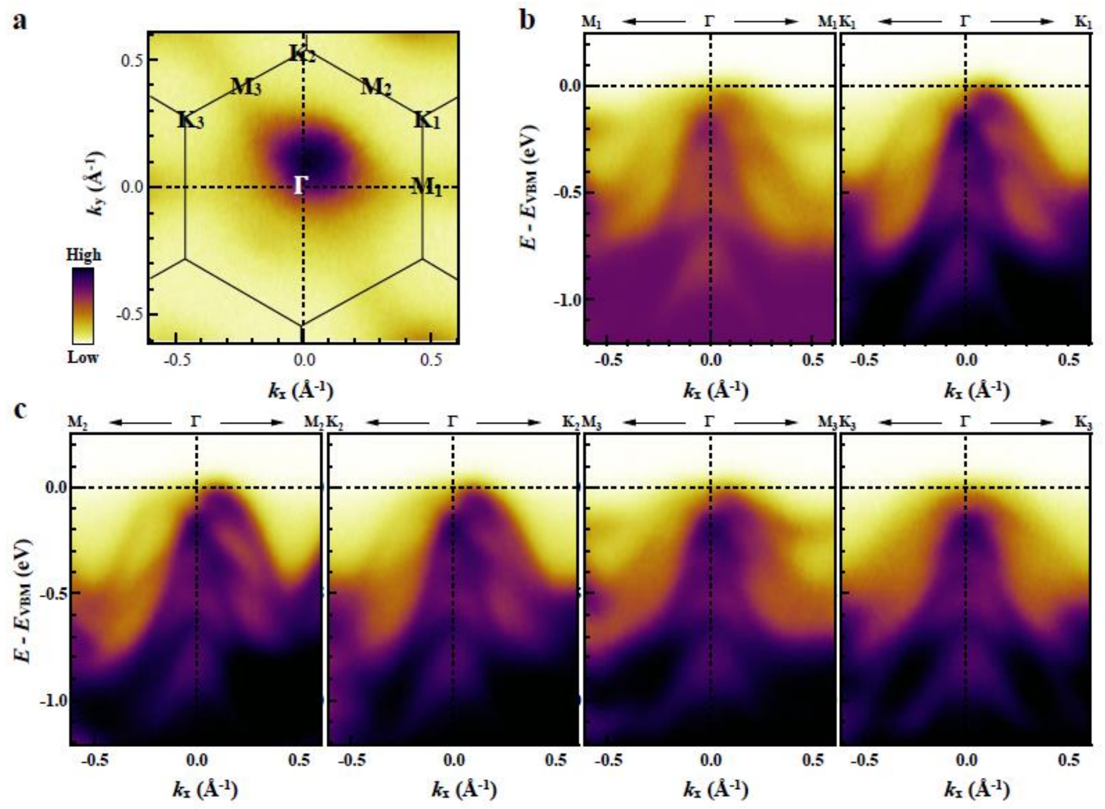

Figure S10

(a) Experimental photoemission signal as a function of in-plane momentum at an energy close to the VBM for a section through the Brillouin zone, showing the special points of the 2D quasi-Brillouin zone. This data can be compared to that of Fig. 3 of the main text but, as for Fig. S9, was now recorded with an excitation photon energy of 118 eV corresponding, according to the calculation of Fig. S4, to placing the three-dimensional Γ point at $k_x = k_y = 0$ in the ARPES maps. (**b**) ARPES data along the M'ΓM and K'ΓK directions in the reciprocal space (in each panel, k_x represents the in-plane momentum in the particular direction shown at the top of the panel). As analysed in detail for the Z point, the dispersion is flatter in the M1 direction and is more pronounced in the other directions.
